# Surgical management of contracted eye socket

**DOI:** 10.1016/j.jobcr.2026.101429

**Published:** 2026-02-27

**Authors:** Eman Mohammed Mahmoud, Ahmed Ali Taha, Essam A. Eltoukhy, Ashraf Elsebaei Mohammed, Ahmed F. Aborady

**Affiliations:** a Plastic Reconstructive Department, Ministry of Health, Cairo, Egypt; b Plastic Reconstructive Department, Faculty of Medicine, Cairo University, Cairo, Egypt; c Ophthalmology Departments, Faculty of Medicine, Cairo University, Cairo, Egypt

**Keywords:** Socket reconstruction, Eye prosthesis, Contracted socket, Anophthalmia

## Abstract

**Background:**

Anophthalmia—the absence of an eye—can significantly impact patients’ psychological well-being, affecting self-confidence, body image, social interactions, and contributing to anxiety and depression. Contracted socket, characterized by reduced orbital volume and forniceal depth, poses a major challenge to fitting an ocular prosthesis. This study evaluates the effectiveness of skin grafts and mucous membrane grafts in forniceal reconstruction.

**Methods:**

This prospective case series included 25 patients with varying degrees of contracted sockets treated between October 2020 and December 2023. Etiologies included congenital anophthalmia, chemical burns, radiotherapy, infection, and trauma. Reconstruction was performed using mucous membrane grafts (MMG) or full-thickness skin grafts (FTSG), according to the severity of socket contraction. Outcomes were evaluated based on the ability to retain a prosthesis and patient satisfaction.

**Results:**

Twenty-four of the 25 patients achieved successful prosthesis retention. Complications were minimal and appropriately managed. Serial dilatation was particularly important in preventing re-contracture, especially in Grade 4 sockets.

**Conclusions:**

Simple reconstructive techniques using MMG and FTSG can restore forniceal depth effectively, achieving favorable cosmetic and functional outcomes while minimizing the need for complex procedures.

## 1 Introduction

Any form of facial disfigurement may lead to profound psychological consequences, especially in societies where visible differences are stigmatized.^1^ Anophthalmia negatively affects mental health by reducing self-esteem, altering body image, and diminishing social confidence.^2^ Congenital conditions such as anophthalmia and microphthalmia can also result in bony orbital hypoplasia and impaired orbitofacial development, making early intervention critical.^3^

Acquired anophthalmia is the leading cause of contracted socket and may follow trauma, evisceration, enucleation, exenteration, chemical burns, inflammatory disease, or infection.^4^ Contracted socket is characterized by reduced orbital volume and shallowing of the fornices, creating difficulty or inability to retain an ocular prosthesis. While contracted sockets were once considered extremely difficult to manage, advances in reconstructive surgery have enabled reliable restoration of fornix depth and prosthesis retention.

Multiple studies have attempted to classify contracted sockets by severity and provide management algorithms, including the widely used Tawfik Classification.^5^ Despite this, high failure rates—particularly in severe or recurrent cases—remain a challenge.

This study aims to evaluate simple yet effective reconstructive techniques to achieve reliable, reproducible outcomes for patients with contracted sockets.

## 2 Patients and methods

This prospective case series was conducted at the Plastic Reconstructive Surgery Department, Cairo University, between October 2020, and December 2023. The study included twenty-five patients presenting with varying degrees of socket contracture who maintained a desire to wear an ocular prosthesis. Ethical approval was obtained from the institutional review board (Approval No: MD-166-2020), and written informed consent was secured from all participants, with additional counseling provided for patients under the age of 18. The study excluded patients with malignant orbital disease, metastasis, or those with sockets following total exenteration.

The severity of socket contraction was graded according to the Tawfik Classification, ranging from Grade 1 (minimal contracture) and Grade 2 (moderate contracture involving one fornix with lid deformity) to Grade 3 (contracted upper and lower fornices with inability to retain a prosthesis and Grade 4 (phimosis of palpebral aperture, irradiated, or recurrent cases).

Preoperative preparation involved photography, a full medical investigation, and, when indicated, culture and sensitivity testing. Additionally, four custom conformers were fabricated for each patient prior to surgery.

**Fig. 1:**
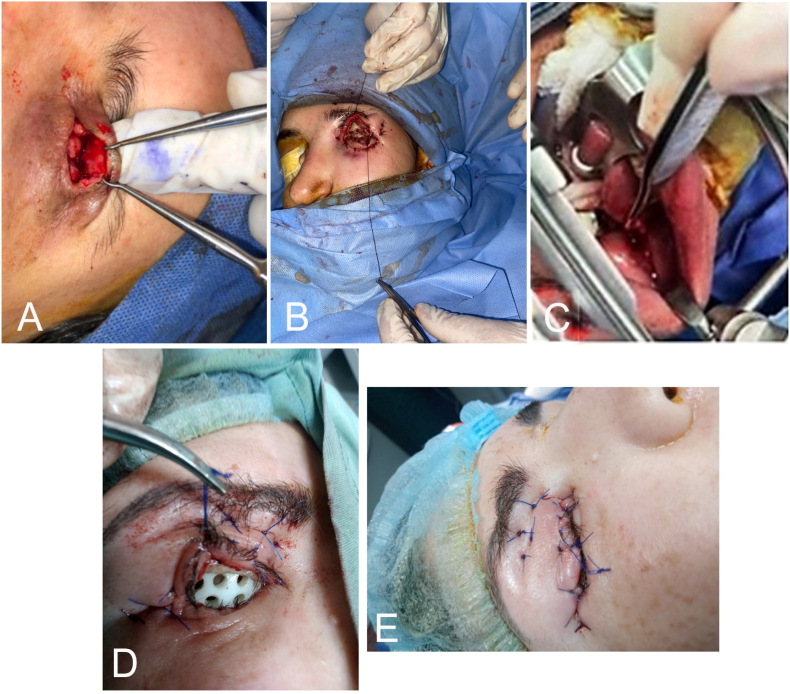
**Surgical technique steps.** (A) Fibrous tissue was dissected and excised. (B) The skin graft was inserted. (C) Mucous membrane (MM) was harvested using Dingman retractors and the graft was fixated. (D) A multi-hole conformer was inserted. (E) Tarsorrhaphy was performed over the conformer.

All surgical procedures were performed under general anesthesia (Fig. 1) Exposure was achieved using a 3/0 Prolene traction suture passed through the tarsal plate followed by the excision of fibrotic conjunctiva. Fornices were then reconstructed using either a Mucous Membrane Graft (MMG) or a Full-Thickness Skin Graft (FTSG). The FTSG was harvested from the lower abdomen and was reserved primarily for Grade 3 and 4 sockets, fixed with minimal "marionette" sutures. Alternatively, the MMG was harvested from the buccal mucosa via a 1.5-cm incision inferior to the Stensen's duct papillae; the donor site was closed with 4-0 Vicryl, and the graft was sutured into the recipient bed using simple absorbable sutures.

**Fig. 2:**
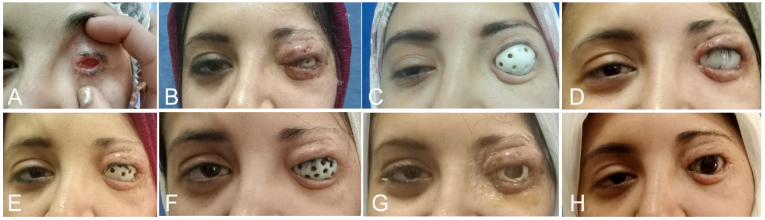
Clinical progression of a 27-year-old female with syndromic microphthalmia and a history of multiple failed left eye socket reconstructions. **(A)** Preoperative presentation; reconstruction was subsequently performed using a full-thickness skin graft (FTSG). **(B)** Clinical appearance was documented one week after the release of tarsorrhaphy sutures. **(C)** The conformer became ill-fitting as a result of postoperative socket shrinkage. **(D**–**F)** Serial dilatation was performed using progressively larger conformers, which were exchanged at four-day intervals. **(G)** The final conformer size was maintained for a duration of two months. **(H)** The patient wore the final ocular prosthesis, which was fabricated in a home-based laboratory.

Following graft placement, a perforated conformer was inserted to mold the fornices and ensure appropriate graft-to-bed contact. A temporary tarsorrhaphy was maintained for four weeks, after which the conformer remained in place for an additional one to two weeks. For severe Grade 4 sockets, serial dilatation proved essential. Using a home-based lab for rapid fabrication, custom conformers (Figure SI) of progressively increasing sizes were utilized immediately upon any detection of early shrinkage or spontaneous displacement (Fig. 2).

A home-based lab facilitated rapid fabrication of a small, tailored conformers critical for timely management. Final prostheses were manufactured in the same lab, with professional polishing performed every six months (Fig. S2).

Postoperative care consisted of twice-daily fluoroquinolone eye drops, nightly terramycin ointment, and a five-day course of systemic broad-spectrum antibiotics. Patients were followed weekly for the first eight weeks and every four months thereafter, during which they were instructed on prosthesis hygiene and handling.

Outcomes were assessed based on the adequacy of the fornices—specifically depth, prosthesis retention, and eyelid contour—as well as patient satisfaction via a 5-point Likert scale. Structured interviews were also conducted to evaluate psychological improvements in social confidence and emotional well-being (Table 3).

## 3 Statistical analysis

Data were managed and analyzed using IBM SPSS Statistics (Version 26.0). All variables were coded prior to analysis, including demographic data, etiology (trauma, infection, chemical burn, congenital, or malignancy/radiotherapy), and socket grade. Reconstruction methods were categorized as MMG, FTSG, or FTSG combined with serial dilatation. Primary outcomes, such as prosthesis retention and fornix adequacy, were coded binarily and summarized using simple proportions.

Continuous variables were expressed as mean ± standard deviation (SD) and range, while categorical variables were summarized as frequencies and percentages. To ensure data integrity, operative logs were verified to confirm the inclusion of 25 consecutive patients, addressing any previous discrepancies. As this study was designed as a single-cohort descriptive case series without a control group, inferential statistical tests and effect size estimations were not applied to avoid over interpretation of descriptive.

Future comparative studies using parallel cohorts or randomized allocation will enable formal statistical hypothesis testing.Data expressed as mean ± SD or percentage.p < 0.05 considered significant.

## 4 Results

The demographic and clinical characteristics of the study population are summarized in Table 1. The study included twenty-five patients, consisting of 15 males and 10 females, with the right socket affected in 14 cases and the left in 11 cases. Regarding the etiology of socket contracture, radiotherapy was the most common cause (n = 10), followed by congenital anophthalmia (n = 7), chemical burns (n = 5), trauma (n = 2), and infection (n = 1). According to the Tawfik Classification, 5 patients presented with Grade 2 contracture, 7 with Grade 3, and 13 with Grade 4. Notably, the Grade 4 cases were characterized by longstanding, recurrent contractures, with patients having been prosthesis-free for 10 to 20 years. Reconstructive strategies were individualized according to contracture severity. Mucous membrane grafts (MMG) were utilized in five patients, while full-thickness skin grafts (FTSG) were employed in seven. The remaining 13 patients, all presenting with severe contractures, underwent FTSG reconstruction combined with a regimen of serial dilatation (Table 2). Within the congenital subgroup, mild cases (n = 4) were treated with MMG, the moderate case (n = 1) received an FTSG, and severe cases (n = 2) required combined FTSG and serial dilatation. It should be clarified that congenital anophthalmia, per se, is not considered contracted socket. However, repeated surgical interventions in cases of congenital anophthalmia can lead to contracted socket so managed and graded as acquired contracted socket.

**Table 1:** Demographic and clinical characteristics of 25 patients with contracted anophthalmic sockets.

Parameter	Subcategory	n (%) or Range
**Gender**	Male	15 (60%)
	Female	10 (40%)
**Age (years)**	Mean ± SD (Range)	33.5 ± 15.73 (7–60)
**Laterality**	Right Socket	14 (56%)
	Left Socket	11 (44%)
**Etiology**	**Acquired**	**18 (72%)**
	└ Post-radiotherapy	10 (40%)
	└ Post-chemical burn	5 (20%)
	└ Post-trauma	2 (8%)
	└ Post-infection	1 (4%)
	**Congenital**	**7 (28%)**
**Tawfik Grading**	Grade 2	5 (20%)
	Grade 3	7 (28%)
	Grade 4	13 (52%)
**Volume Replacement**	No implant	23 (92%)
	Orbital implant present	2 (8%)
**Surgical History**	First-time reconstruction	12 (48%)
	Recurrent/Long-standing failure∗	13 (52%)

**Table 2:** Reconstructive methods Stratified by Tawfik socket grade.

Tawfik Grade	Severity	Reconstructive Method	n (%)
**Grade 2**	Mild	Mucous Membrane Graft (MMG)	5 (20%)
**Grade 3**	Moderate	Full-Thickness Skin Graft (FTSG)	7 (28%)
**Grade 4**	Severe	FTSG + Serial Dilatation	13 (52%)
**Total**			**25 (100%)**

**Table 3:** Outcomes of socket reconstruction by technique: Wear success and patient satisfaction (n = 25).

Reconstruction Technique	No. Of Patients	Prosthesis Retention Success	Adequacy of Fornices	Mean Satisfaction Score (1–5)	Follow-up Duration
**Mucous Membrane Graft (MMG)**	5	5/5 (100%)	5/5 (100%)	4.8	Minimum 12 months
**Full-Thickness Skin Graft (FTSG)**	7	7/7 (100%)	7/7 (100%)	4.7	Minimum 12 months
**FTSG + Serial Dilatation**	13	12/13 (92.3%)	12/13 (92.3%)	4.0	Minimum 12 months
**Total**	25	24/25 (96%)	24/25 (96%)	4.36	Minimum 12 months

Donor site morbidity was minimal; all MMG donor sites healed uneventfully. FTSG donor sites also demonstrated favorable healing, with the exception of one mild infection in a diabetic patient that resolved with local wound care.

Successful prosthesis retention was achieved in 24 of 25 patients (96%). Patient satisfaction, quantified via a 5-point Likert scale, reflected high overall success: 15 patients (60%) reported being "very satisfied," six (24%) "satisfied," and three (12%) "neutral." A single patient (4%) reported dissatisfaction, which was primarily attributed to recurrent mucormycosis and a persistent medial wall infection (Table 4).

**Table 4:** Patient satisfaction according to the Likert scale.

Satisfaction Level	Number of Patients (n)	Percentage (%)
Very Satisfied	15	60%
Satisfied	6	24%
Neutral	3	12%
Dissatisfied	1	4%
Very Dissatisfied	0	0%
**Total**	**25**	**100%**

**Fig. 3:**
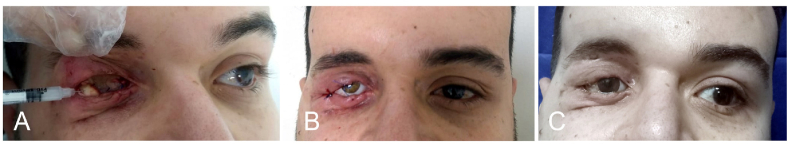
Management of a keloid-like scar in the periorbital region. **(A)** The clinical presentation of the keloid-like scar was recorded. **(B)** An intralesional injection of corticosteroid (10 mg/ml triamcinolone acetonide) was administered. **(C)** Lateral tarsorrhaphy was performed over the ocular prosthesis to provide structural support. **(D)** Clinical appearance following the release of the tarsorrhaphy demonstrated a stable and well-positioned prosthesis.

Postoperative complications were documented and managed on an individual basis (Table 5). Two patients developed keloid-like cicatrization resulting in prosthesis extrusion (Fig. S3). These complications were successfully resolved using a combination of intralesional corticosteroid injections and a temporary tarsorrhaphy maintained for three weeks (Fig. 3).

**Table 5:** Complications by reconstruction technique with overall incidence.

Complication	MMG (n = 5)	FTSG (n = 7)	FTSG + Serial Dilatation (n = 13)	Overall (n = 25)
Keloid-like scarring	0 (0%)	0 (0%)	2 (15.4%)	2 (8.0%)
Early shrinkage (post-tarsorrhaphy)	0 (0%)	0 (0%)	13 (100%)∗	13 (52.0%)∗
Donor site infection	0 (0%)	1 (14.3%)	0 (0%)	1 (4.0%)
Recurrent mucormycosis infection	0 (0%)	0 (0%)	1 (7.6%)	1 (4.0%)
**Total patients with ≥1 complication**	**0 (0%)**	**1 (14.3%)**	**13 (100%)**	**14 (56.0%)**

Early socket shrinkage was observed in several cases following the release of the initial tarsorrhaphy; however, these were effectively resolved via serial dilatation (Fig. 2).

A significant complication occurred in one patient who presented with recurrent mucormycosis and disruption of the medial orbital wall. Despite these structural challenges, the patient was able to successfully wear a large, stable, open-ring conformer, which was utilized to facilitate regular irrigation of the socket (Fig. S4). However, due to the underlying clinical instability and the risk of further infection, definitive intervention was postponed."

Minor complications included a mild wound infection at the full-thickness skin graft (FTSG) donor site in one diabetic patient. This was successfully managed with local wound care and oral antibiotics, without long-term morbidity."

The study protocol required a minimum follow-up duration of 12 months, during which patients underwent routine prosthesis polishing at six-month intervals to ensure consistent clinical conditions."

**Fig. 4:**
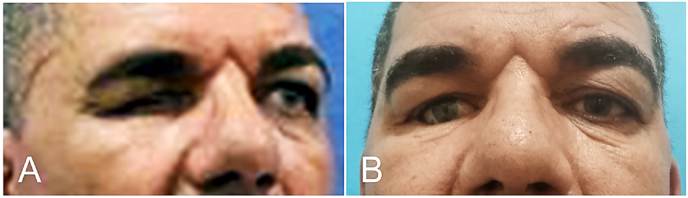
A 50-year-old male with a history of right eye evisceration following trauma at age 7. **(A)** Preoperative presentation showed lower eyelid shelving and prosthesis instability, which was classified as a Grade 2 contracted socket. Reconstruction was performed using a buccal mucosal graft. **(B)** Clinical outcome was assessed 4 months postoperatively following a 4-week period of temporary tarsorrhaphy; the final prosthesis was successfully fitted to the patient.

**Fig. 5:**
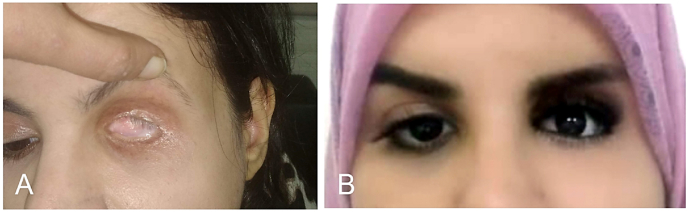
**Surgical management of a Grade 4 contracted socket in a previously irradiated field. (A)** Preoperative presentation of a 33-year-old female with a left anophthalmic socket following childhood exenteration and adjuvant radiotherapy. The patient presented with a Grade 4 contracture and a history of long-term prosthetic non-compliance. Reconstruction was achieved via a full-thickness skin graft (FTSG) followed by a postoperative regimen of serial dilatation. **(B)** Clinical outcome at one year postoperatively, demonstrating successful socket expansion and stable prosthesis retention, artificial eyelashes were applied to the prosthesis to enhance ocular symmetry and the overall cosmetic outcome.

The clinical presentations and postoperative courses are illustrated in Fig. 1, Fig. 2, Fig. 3, Fig. 4, Fig. 5.

Supplementary Fig. S1 Through S7+ A Strobe flow chart:

## 5 Discussion

This research marks a pivotal advancement in treating contracted anophthalmia, especially for severe (Grade 4) cases once considered inoperable, previously referred to malignant contracted socket. The authors successfully challenge this conventional wisdom, demonstrating that meticulous surgical technique combined with structured follow-up can indeed yield positive patient outcomes. This finding is profoundly significant, offering renewed hope and a more proactive treatment paradigm for patients previously deemed beyond repair.

The study's optimized grafting strategies are particularly noteworthy. Their discovery that serial dilatation with full-thickness skin grafts (FTSG) is highly effective for progressive socket expansion, particularly in historically challenging cases, provides surgeons with a powerful new therapeutic tool.

Furthermore, the identification of the lower abdomen as a superior donor site for FTSG represents a practical and impactful innovation. The ability to harvest a larger graft with minimal donor site complications offers a substantial advantage over traditional postauricular sites, potentially leading to improved aesthetic and functional results for patients. The subtle yet critical detail of employing a graft size nearly identical to the defect for precise tensioning highlights the meticulous nature of their surgical technique and its positive influence on graft integration, contrasting with other studies that recommend a graft 50% larger than the defect.^6^ Contrary to concerns raised in other studies regarding chronic discharge from FTSG, all reconstructed sockets in our cohort remained dry, with patients adhering to a cleaning regimen every two weeks.

The study highlights the critical importance of precise patient selection in surgical outcomes. By strategically reserving mucous membrane grafting (MMG) for less severe Grade 2 contracted sockets, the researchers achieved a remarkable 100% success rate. This approach stands in sharp contrast to other research, such as Aryasit et al.,^7^ where MMG was applied to more severe cases with a comparatively lower success rate of 80%. These findings strongly suggest that a more conservative and targeted application of MMG can significantly enhance patient outcomes and reduce associated complications.

The refinement of the tarsorrhaphy technique, advocating for simple tarsorrhaphy over bolster-supported methods, reflects a clear commitment to patient comfort and accelerated recovery. This preference stems from observations of faster resolution of lid swelling and reduced complications, aligning well with existing literature^8^ and reinforcing best practices.

The capability to fabricate **custom prostheses in-house** not only reduces costs but also significantly improves fit and cosmetic outcomes, directly enhancing patient satisfaction and overall well-being. Finally, the successful management of secondary complications, such as socket scarring and keloid formation, using established protocols demonstrates a thorough approach to post-operative care, anticipating and effectively addressing potential challenges.

Beyond the surgical advancements, the comprehensive patient care protocol warrants emphasis. This protocol, encompassing patient education, psychosocial support, and in-house prosthesis fabrication, addresses the holistic needs of individuals with contracted sockets. The low incidence of re-contraction, attributed to patient education on hygiene and prosthesis care, underscores the critical importance of patient adherence.

## 6 Strengths of the study

A key strength of this study is the consistency of surgical technique, postoperative regimen, and follow-up, as all procedures were performed by the same surgeon following one unified algorithm. The cohort also reflects a real-world sample that includes congenital cases, trauma, burns, and post-radiotherapy sockets, supporting generalizability. Additionally, the structured dilatation protocol and immediate access to custom conformer fabrication represent practical innovations that likely contributed to the high prosthesis retention rate.

## 7 Limitations in the context of literature

This study, similar to other case series in the literature^356^, is limited by lack of a control group and reliance on descriptive outcomes rather than objective metrics such as standardized psychological scores or instrument-based fornix measurements. Although the reported success rate is high, the absence of quantitative functional scales or patient-reported outcome measures limits the ability to compare with other studies directly. Nonetheless, the consistent improvement across nearly all patients provides clinically meaningful evidence supporting this treatment algorithm.

## 8 Future directions

Future research should include comparative studies evaluating MMG and FTSG against other techniques such as amniotic membrane grafts, dermis-fat grafts, and tissue-engineered constructs. Longer follow-up with objective imaging or measurement tools could provide more quantitative assessments of graft integration and fornix stability. Incorporating validated psychological assessment tools would also better quantify the psychosocial benefits associated with prosthesis rehabilitation.

## 9 Summary

In summary, this study supports the use of MMG, FTSG, and structured serial dilatation as effective methods for managing contracted sockets across a range of severities. Our findings differed from in several aspects from the previously cited studies, and highlight the importance of graft selection, conformer support, and vigilant postoperative follow-up in achieving stable, functional, and esthetically acceptable outcomes.

## Patient consent

Written informed consent was obtained from all patients.

## Patient's/guardian's consent

Written informed consent for participation and for the use of clinical photographs was obtained from all patients or their legal guardians prior to inclusion in the study.

## Ethical approval

Approved by the Cairo University Ethics Committee (MD-166-2020).

## Ethical clearance

The study has been carried out in accordance with The Code of Ethics of the World Medical Association (Declaration of Helsinki) for experiments involving humans. This study protocol was approved by Research Ethics Committee of the Faculty of Medicine, Cairo University (Approval No: MD-166-2020).

## Author agreement

All authors approved the final manuscript.

## Funding

None.

## Declaration of competing interest

The authors declare that they have no known competing financial interests or personal relationships that could have appeared to influence the work reported in this paper.
