# Supplementary material for: Surgical management of contracted eye socket
Source: J Oral Biol Craniofac Res. 2026 Feb 27;16(2):101429. doi: 10.1016/j.jobcr.2026.101429 (PMC12966714; doi:10.1016/j.jobcr.2026.101429)
Supplement: Multimedia component 1 [file mmc1.docx]

**Supplementary Figures**


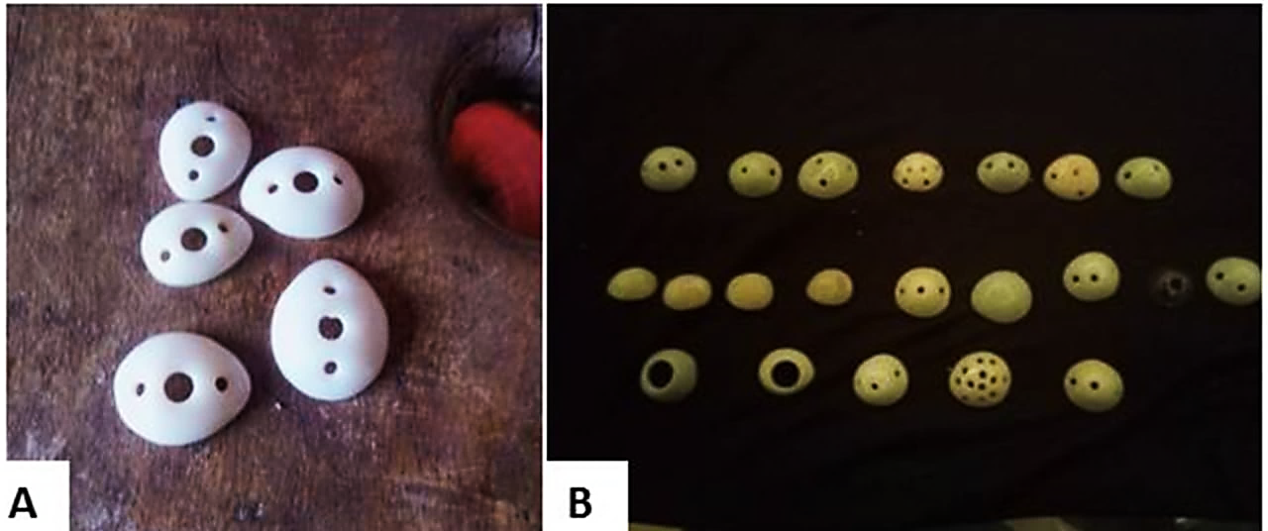


**Figure S1**. Multiple sized conformers.

**
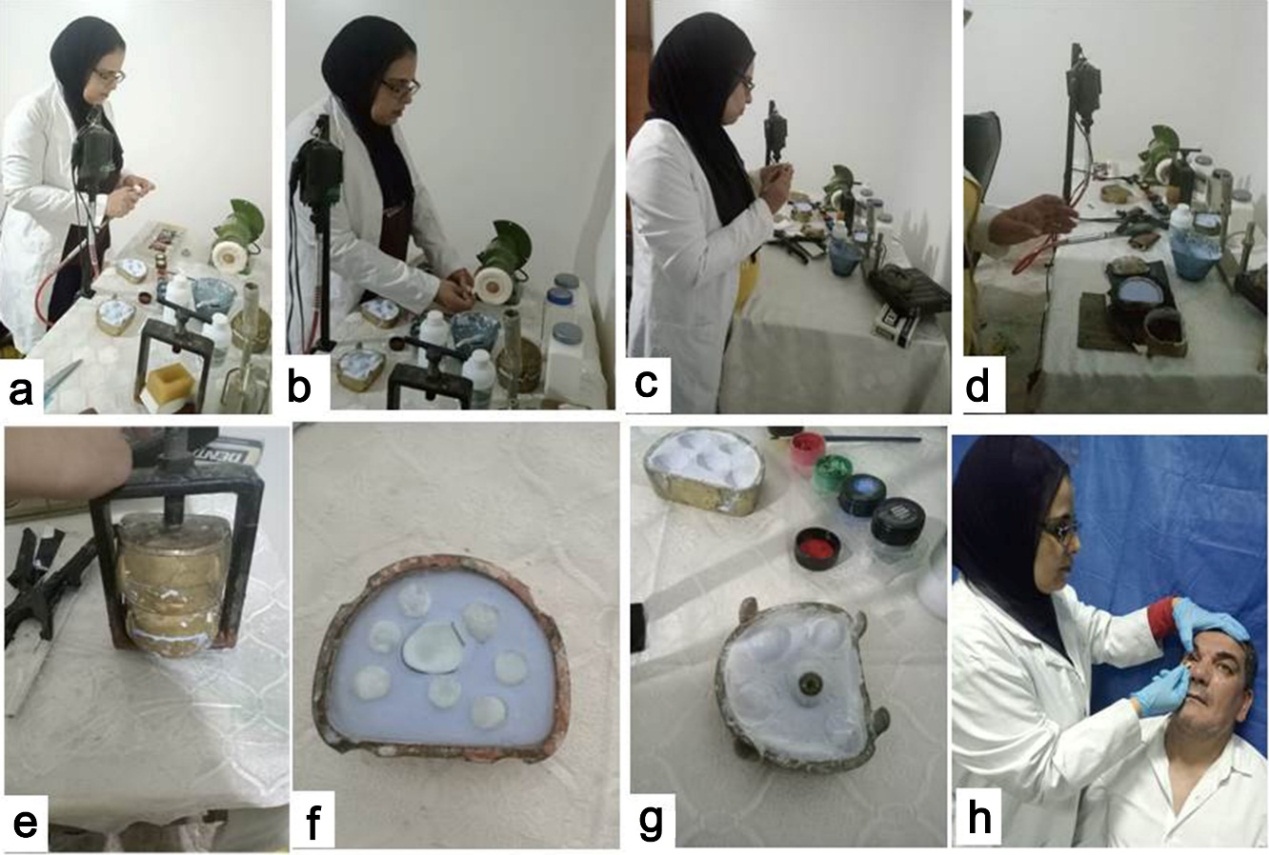
**

**Figure S2***.* A home-based lab with some steps of prosthesis making (a-h).

**
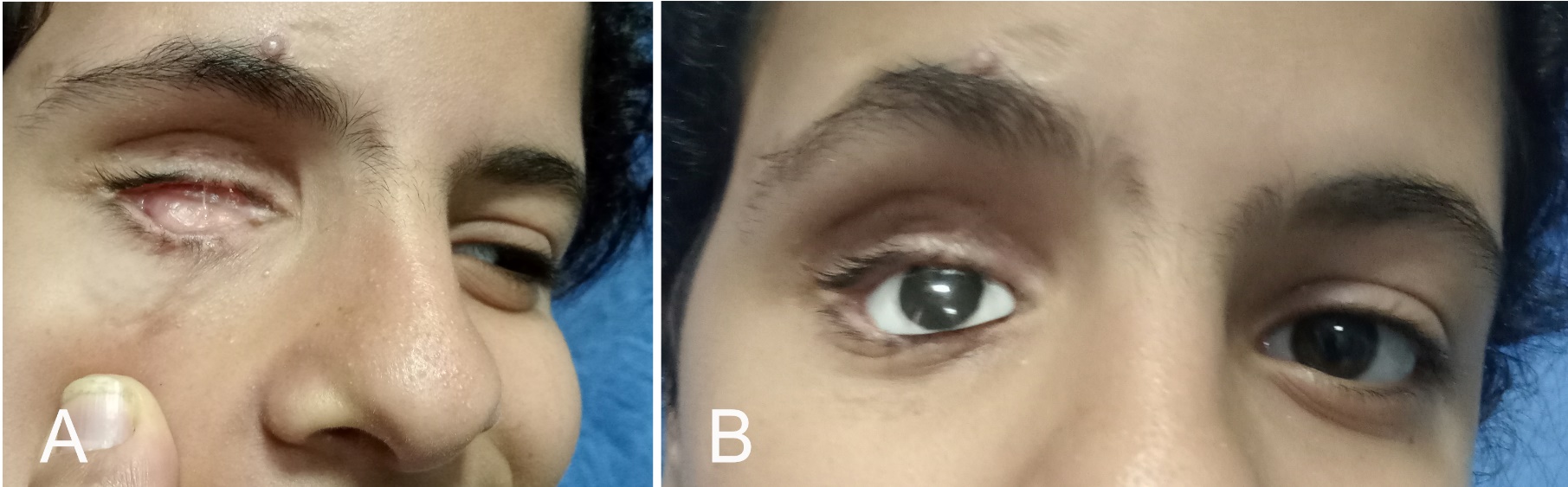
**

**Figure S3***.* Postoperative complications and clinical findings. **(A)** Detailed view demonstrating the formation of a keloid-like scar at the surgical site. **(B)** Clinical photograph showing prosthesis extrusion.

**
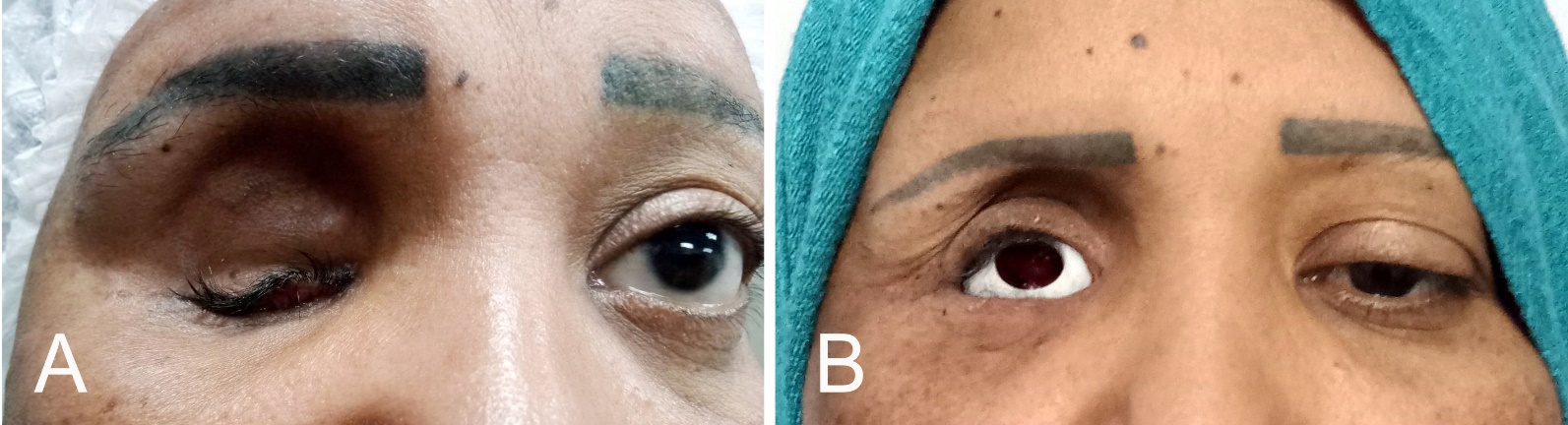
**

**Figure S4***.* Management of a severely contracted right anophthalmic socket following mucormycosis. **(A)** Preoperative presentation showed severe socket contracture and volume deficit secondary to invasive mucormycosis. **(B)** Postoperative maintenance was achieved using an open-ring conformer to preserve the reconstructed fornices while allowing for drainage and topical medication.

**
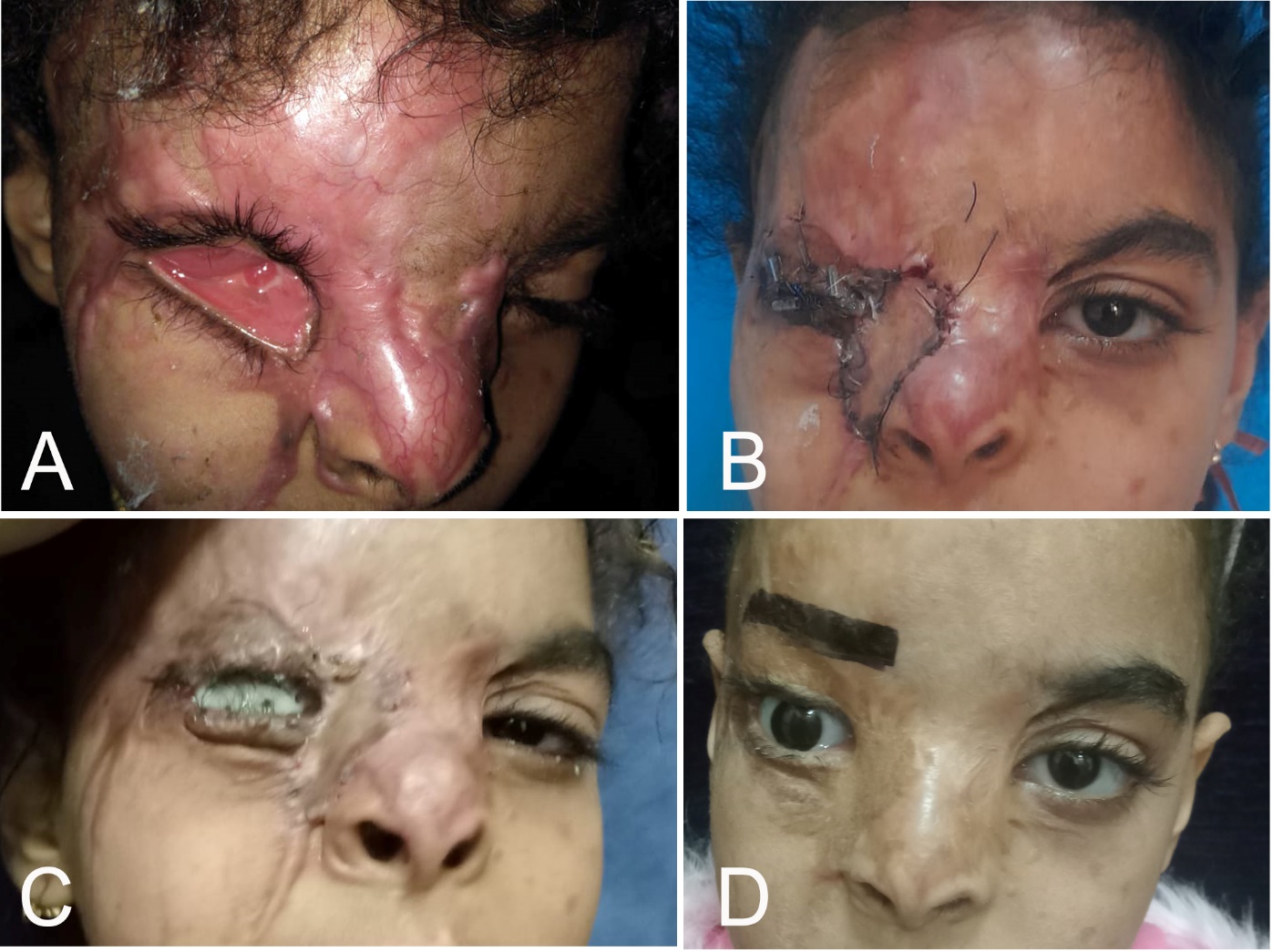
**

**Figure S5***.* **(A)** An 8-year-old female child presented following a chemical burn. **(B)** The lower and upper eyelids were released, and coverage was achieved using a full-thickness skin graft (FTSG). **(C)** The upper and lateral fornices were reconstructed, and a conformer was placed. **(D)** The final result was captured one year postoperatively with the patient wearing a permanent ocular prosthesis.

*
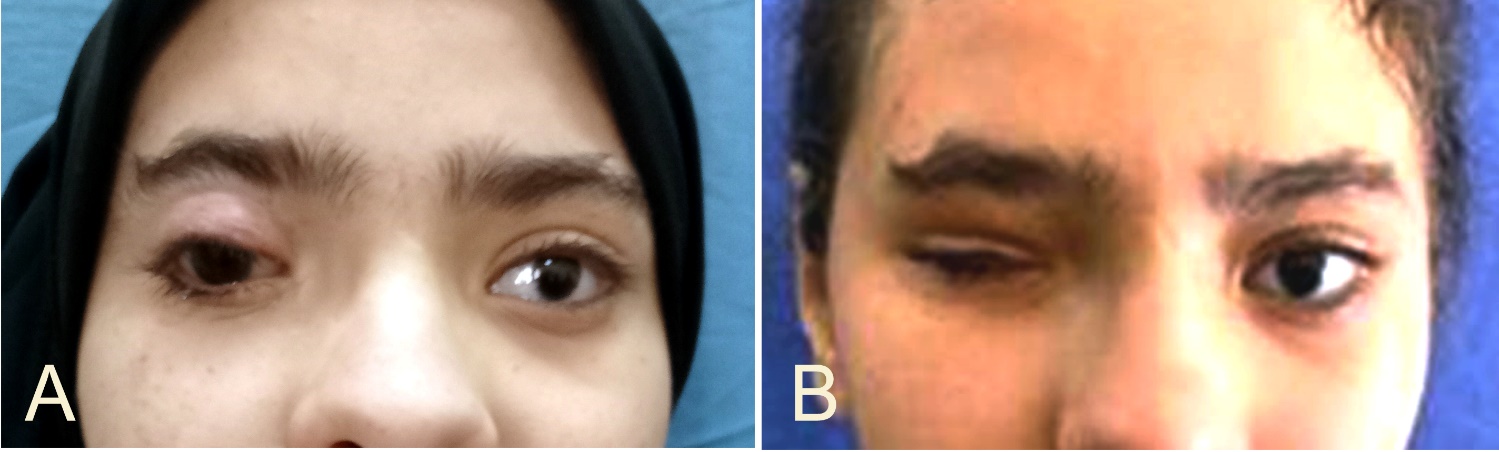
*

**Figure S6***.* A 21-year-old female patient presented with congenital microphthalmia and had not received early expansion. Two previous surgical attempts had been unsuccessful. The patient presented with an obliterated upper fornix and was diagnosed with a grade 2 contracted socket. Reconstruction was performed using buccal mucosa. A tarsorrhaphy was maintained for five weeks, and a prosthesis was fitted four months postoperatively.


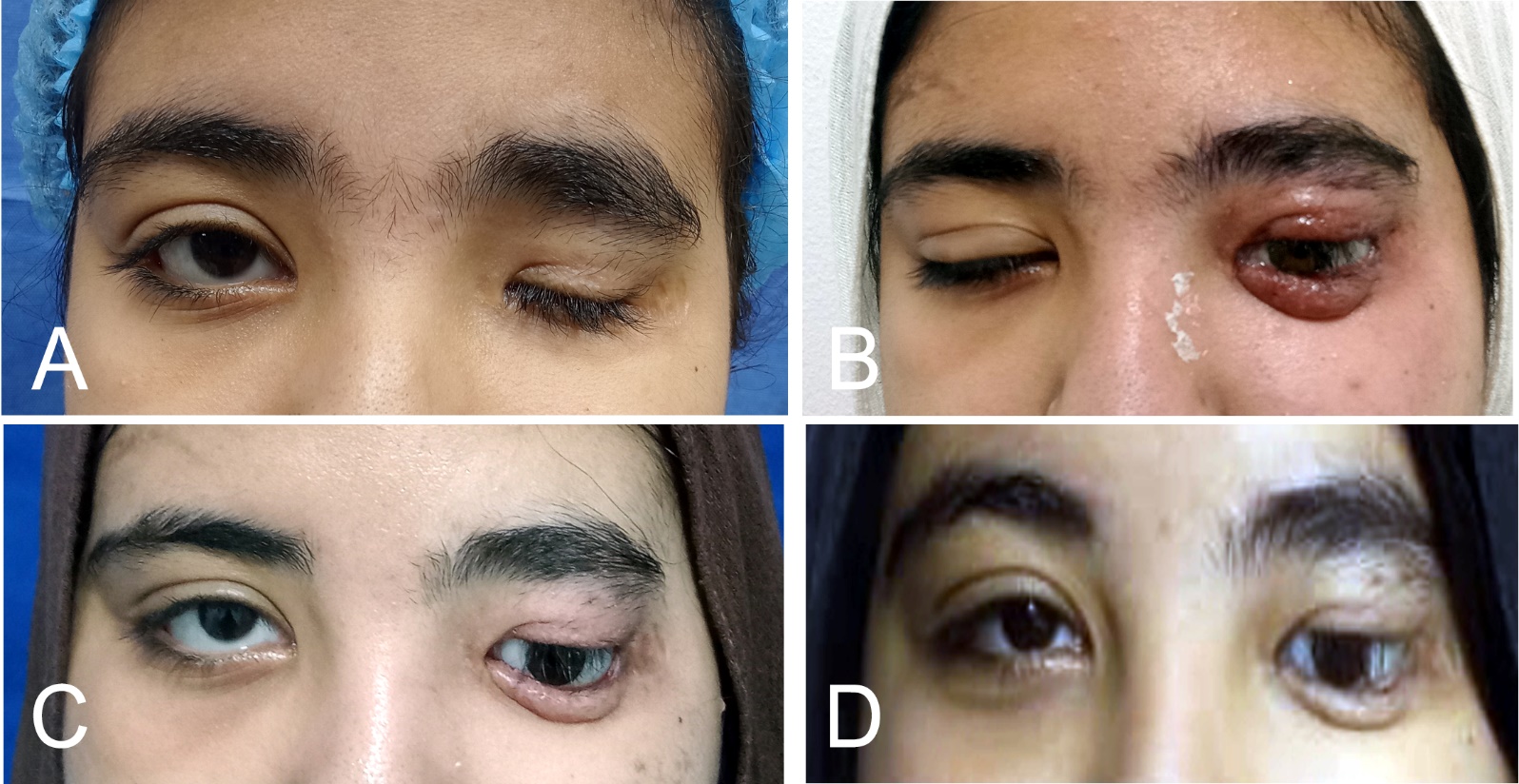


**Figure S7***.* **(A)** A 16-year-old female presented with left microphthalmia. **(B)** The appearance was captured following the release of a tarsorrhaphy. **(C)** The patient was fitted with a temporary prosthesis. **(D)** The results **were** evaluated at six months and **(E)** one year postoperatively.
